# Draft Genome Sequence of *Desulfuromonas acetexigens* Strain 2873, a Novel Anode-Respiring Bacterium

**DOI:** 10.1128/genomeA.01522-16

**Published:** 2017-03-02

**Authors:** Krishna P. Katuri, Mads Albertsen, Pascal E. Saikaly

**Affiliations:** a Biological and Environmental Science and Engineering (BESE) Divison, Water Desalination and Reuse Center (WDRC), King Abdullah University of Science and Technology (KAUST), Thuwal, Saudi Arabia; b Department of Chemistry and Bioscience, Center for Microbial Communities, Aalborg University, Aalborg East, Denmark

## Abstract

Here, we report the draft genome sequence of *Desulfuromonas acetexigens* strain 2873, which was originally isolated from digester sludge from a sewage treatment plant in Germany. This bacterium is capable of anode respiration with high electrochemical activity in microbial electrochemical systems. The draft genome contains 3,376 predicted protein-coding genes and putative multiheme c-type cytochromes.

## GENOME ANNOUNCEMENT

*Desulfuromonas acetexigens* strain 2873 (DSM 1397), first isolated from digester sludge from the Göttingen, Germany, sewage treatment plant (1), is a Gram-negative, non-spore-forming anaerobic bacterium that is capable of chemoorganotrophic growth on acetate as the energy and carbon source with elemental sulfur, fumarate, or malate as electron acceptors (1). Recently, its high abundance in mixed-culture anodic biofilms of current-generating microbial electrochemical systems (MESs) (2–4) suggests its role in current generation. Pure culture studies in MESs confirmed that *D. acetexigens* strain 2873 is capable of extracellular electron transfer (EET) to the anode, which serves as the electron acceptor, producing high peak current densities of 7 to 9 A/m^2^ in a very short growth period with acetate as the electron donor (5, 6). However, our understanding of how *D. acetexigens* strain 2873 transfers electrons to and from electrodes is lacking, and sequencing and analysis of its genome could help elucidate its EET mechanism(s).

Here, we assembled and annotated a draft genome of *D. acetexigens* strain 2873. Genomic DNA extracted from strain 2873 was prepared for genome sequencing using the Illumina Nextera DNA library preparation kit (Illumina, Inc.), following the manufacturer’s instructions. Sequencing was performed on Illumina MiSeq instruments at DNASense Aps (Aalborg, Denmark) using the MiSeq reagent kit version 3 (Illumina, Inc.), yielding 2.7 million paired-end reads. Raw reads were quality trimmed using Trimmomatic version 0.36 (7), and the trimmed paired-end Illumina reads were *de novo* assembled using SPAdes version 3.7.1 (8). The genome assembly produced 41 contigs with a total length of 3,683,125 bp (G+C content, 60.3%) and an *N*_50_ of 301,105 bp.

The assembled genome was annotated using Prokka version 1.11 (9), with the genome of *Geobacter sulfurreducens* PCA (NC_002939) (10), a well-known model organism in the field of microbial electrochemistry, used as a reference for annotation. Annotation revealed two rRNA operons, six rRNA genes, 56 tRNA genes, and 3,376 predicted protein-coding genes. Among the predicted protein-coding genes, those encoding for c-type cytochromes are of primary interest. Based on the methodology of Methé et al. (10), all predicted proteins containing putative heme-binding motifs were screened using PROSITE (11) with the following motifs: PS51007, cytochrome c family; PS51008, multiheme cytochrome c family; PS51009, cytochrome c class II; and PS51010, cytochrome f family. In addition, the proteins were annotated using the genome of *G. sulfurreducens* PCA (NC_002939) as a reference to ensure consistent cytochrome annotation. Cytochrome annotation revealed the presence of 39 putative multiheme c-type cytochromes, including the outer membrane c-type cytochromes, OmcS and OmcE, which have been reported to play a key role in current generation in* G. sulfurreducens* (12). Further analyses of functional annotations, metabolic pathways, and comparative genomics are under way and will be included in a future publication.

### Accession number(s).

The *D. acetexigens* genome sequence has been deposited in the European Nucleotide Archive under the accession numbers FOJJ01000001 to FOJJ01000041.

## References

[1] Finster K, Bak F, Pfennig N 1994 *Desulfuromonas acetexigens* sp. nov., a dissimilatory sulfur-reducing eubacterium from anoxic freshwater sediments. Arch Microbiol 161:328–332. doi:10.1007/BF00303588.

[2] Ishii S, Suzuki S, Norden-Krichmar TM, Nealson KH, Sekiguchi Y, Yuri AG, Orianna B 2012 Functionally stable and phylogenetically diverse microbial enrichments from microbial fuel cells during wastewater treatment. PLoS One 7:e30495. doi:10.1371/journal.pone.0030495. 22347379 PMC3274515

[3] Katuri KP, Kamireddy S, Saikaly PE, Leech D 2013 Role of tailor made carbon surfaces to anchor and enhance the electrochemical properties of electro-active bacterial biofilms, Abstract O10.3. Proceedings of the Second International Conference on Water Research, Singapore.

[4] Ketep SF, Bergel A, Bertrand M, Achouak W, Fourest E 2013 Lowering the applied potential during successive scratching/reinoculation improves the performance of microbial anodes for microbial fuel cells. Bioresour Technol 127:448–455. doi:10.1016/j.biortech.2012.09.008. 23138069

[5] Bandar KB 2014 Electromicrobiology of dissimilatory sulfur reducing bacterium *Desulfuromonas**acetexigens*. MSc thesis King Abdullah University of Science and Technology, Thuwal, Saudi Arabia.

[6] Katuri KP, Saikaly PE 2016 Electrochemical behavior of a novel electricigen and its whole genome sequence, Abstract 116. Third Asia-Pacific Meeting of the International Society for Microbial Electrochemistry and Technology, Busan, Korea.

[7] Bolger AM, Lohse M, Usadel B, Planck M, Plant M, Mühlenberg A 2014 Trimmomatic: a flexible trimmer for Illumina sequence data. Bioinformatics 30:2114–2120. doi:10.1093/bioinformatics/btu170. 24695404 PMC4103590

[8] Bankevich A, Nurk S, Antipov D, Gurevich AA, Dvorkin M, Kulikov AS, Lesin VM, Nikolenko SI, Pham S, Prjibelski AD, Pyshkin AV, Sirotkin AV, Vyahhi N, Tesler G, Alekseyev MA, Pevzner PA 2012 SPAdes: a new genome assembly algorithm and its applications to single-cell sequencing. J Comput Biol 19:455–477. doi:10.1089/cmb.2012.0021. 22506599 PMC3342519

[9] Seemann T 2014 Prokka: rapid prokaryotic genome annotation. Bioinformatics 30:2068–2069. doi:10.1093/bioinformatics/btu153. 24642063

[10] Methé BA, Nelson KE, Eisen JA, Paulsen IT, Nelson W, Heidelberg JF, Wu D, Wu M, Ward N, Beanan MJ, Dodson RJ, Madupu R, Brinkac LM, Daugherty SC, DeBoy RT, Durkin AS, Gwinn M, Kolonay JF, Sullivan SA, Haft DH, Selengut J, Davidsen TM, Zafar N, White O, Tran B, Romero C, Forberger HA, Weidman J, Khouri H, Feldblyum TV, Utterback TR, Van Aken SE, Lovley DR, Fraser CM 2003 Genome of *Geobacter sulfurreducens*: metal reduction in subsurface environments. Science 302:1967–1969. doi:10.1126/science.1088727. 14671304

[11] Sigrist CJA, De Castro E, Cerutti L, Cuche BA, Hulo N, Bridge A, Bougueleret L, Xenarios I 2013 New and continuing developments at PROSITE. Nucleic Acids Res 41:D344–D347. doi:10.1093/nar/gks1067. 23161676 PMC3531220

[12] Kim BC, Postier BL, DiDonato RJ, Chaudhuri SK, Nevin KP, Lovley DR 2008 Insights into genes involved in electricity generation in *Geobacter sulfurreducens* via whole genome microarray analysis of the OmcF-deficient mutant. Bioelectrochemistry 73:70–75. doi:10.1016/j.bioelechem.2008.04.023. 18538641

